# The relationship between physical exercise and Chinese college students’ career decision-making difficulties: a chain mediating model of psychological capital and employment attitude

**DOI:** 10.3389/fpsyg.2025.1693354

**Published:** 2025-11-21

**Authors:** Yuan Fang, Tingting Xu, Siyu Hong, Changquan Li, Qunqun Sun, Hao Gou, Zhongju Wen

**Affiliations:** 1 School of Physical Education, Qiannan Normal University for Nationalities, Duyun, Guizhou, China; 2 Library, Qiannan Normal University for Nationalities, Duyun, Guizhou, China; 3 Graduate School, Shandong Sport University, Jinan, Shandong, China

**Keywords:** physical exercise, career decision-making difficulties, psychological capital, employment attitude, Chinese college students

## Abstract

**Objectives:**

By exploring the intrinsic connection between physical exercise and career decision-making difficulties (CDMD), this study aims to provide new theoretical insights and practical strategies for understanding and improving career decision-making among college students, as well as enhancing their decision-making capabilities.

**Methods:**

Non-probability convenience sampling was employed to administer an online questionnaire to college students at two universities in Guizhou Province, China. To validate the research hypotheses, a structural equation model was constructed. The bootstrap method facilitated 5,000 repeated random samples, with a significant mediating effect indicated if the 95% confidence interval did not include 0.

**Results:**

(1) Physical exercise exhibits a significant negative association with CDMD. (2) Psychological capital serves as a mediator in the relationship between physical exercise and CDMD. (3) A chained mediating pathway exists between physical exercise and CDMD, operating through psychological capital and employment attitudes. (4) Urban–rural differences exert a moderating effect on the “physical exercise → psychological capital → CDMD” path.

**Conclusion:**

This study reveals that physical exercise is associated with college students’ CDMD through both the single mediating effect of psychological capital and the chain mediating effect of psychological capital and employment attitudes. Physical exercise demonstrates significant potential for alleviating CDMD among college students. By constructing a chain mediation model, this study addresses the research gap regarding the mechanisms linking physical exercise to CDMD, laying a theoretical foundation for future empirical studies on using physical exercise as an intervention for CDMD. This model represents an innovative attempt to explore multiple pathways for alleviating college students’ CDMD, while also expanding the application of the psychological capital theory in the field of career development. The model established in this study can be integrated into physical education and vocational education curricula in higher education, thereby enhancing college students’ career decision-making capabilities. It provides a theoretical basis and practical pathways for innovating relevant courses. Future studies can build on this research to further optimize intervention strategies that utilize physical exercise to alleviate CDMD, addressing existing limitations in the research.

## 1 Introduction

As higher education in China continues to develop and expand its enrollment capacity, employment issues for college students have become increasingly severe and have garnered significant social concern (Zhou et al., 2024). The key to improving the quality and stability of college graduates’ employment lies in career decision-making. This is because the core objective of career decision-making models is to reduce uncertainty through rational analysis of career information, thereby achieving a high degree of fit between individuals and careers (Gati and Kulcsár, 2021). Career decision-making difficulties (CDMD) refer to the various challenges individuals face throughout the career decision-making process (Anghel and Gati, 2019). Gati et al. (1996) categorized these difficulties into three dimensions: Lack of Readiness, Lack of Information, and Inconsistent Information. At the psychological level, these dimensions can manifest as unclear career goals, an inability to rationally evaluate career options, career decision-making anxiety, and self-doubt regarding one’s decision-making abilities. Behaviorally, they may present as avoidance of career decision-making and evasion of preparations related to it (Gati et al., 1996; Amir and Gati, 2006; Anghel and Gati, 2019; Du and Long, 2006; Lam and Santos, 2018). Additionally, CDMD are closely linked to negative emotions such as anxiety and depression, and prolonged difficulties may negatively impact college students’ mental health over time (Gati and Kulcsár, 2021; Otu and Onyishi, 2024; Jia et al., 2025). According to a study by Willner et al. (2015), the level of CDMD among Chinese college students is significantly higher than that among research samples from other countries. Chinese college students face difficulties in making career decisions, according to research by Wang et al. (2023). A comparatively large percentage of them fall into the “undecided” category, which indicates major obstacles in their decision-making processes. According to a study by Song (2025), the difficulties Chinese college students encounter while making professional decisions may be categorized into four groups: biases in the perception of the external environment, biases in the acquisition of job information, biases in the formulation of career goals, and biases in self-perception.

Although researchers have extensively studied interventions for CDMD, the most common approach involves using career intervention courses to address these issues (Fouad et al., 2009; Lam and Santos, 2018). Relevant studies have confirmed that career intervention courses exert a positive effect on CDMD (Gu et al., 2020; Komarraju et al., 2014). Research on vocational intervention programs is significant, but it also reveals a limitation: the approaches to improvement tend to be relatively homogeneous. This homogeneity can adversely affect both individual employment and social development, prompting researchers to explore more effective strategies to alleviate CDMD. Recent evidence suggests that physical exercise has a positive impact on college students’ career decision-making self-efficacy (Fang et al., 2025). However, due to the limited number of existing studies, further evidence is needed to support the positive role of physical exercise in individuals’ career decision-making. Thus, the present study will focus on investigating the underlying mechanisms that associate physical exercise with CDMD. By exploring the intrinsic connection between physical exercise and CDMD, this study aims to provide new theoretical insights and practical strategies for understanding and improving career decision-making among college students, as well as enhancing their decision-making capabilities. In the present study, physical exercise is defined as a type of planned, structured, and repetitive physical activity conducted for the purpose of enhancing or maintaining physical fitness (World Health Organization, 2010).

Recent studies have begun to explore the relationship between exercise and career decisions, offering some intriguing insights. For example, Kim (2018) found that physical self-efficacy and body image, key benefits of engaging in physical exercise, positively influence college students’ career decision-making. Additionally, in a follow-up study, Kim (2019) demonstrated that college students who participated in sports activities had significantly higher levels of career decision-making capabilities than students who did not participate in such activities. Furthermore, Fang et al. (2025) revealed a positive relationship between college students’ physical exercise levels and their career decision self-efficacy. Similarly, Wang et al. (2024) discovered that addressing the basic psychological needs of college students through exercise can enhance their career adaptability. Overall, existing studies have several limitations. Researchers exploring the relationship between physical exercise and career decision-making have primarily focused on improving a specific competency and enhancing self-efficacy. However, there is a gap in studies specifically addressing the link between physical exercise and CDMD. Furthermore, except for Wang et al. (2024), whose study adopted an intervention experimental design, all other studies have drawn conclusions based on cross-sectional surveys.

The introduction of other strongly correlated variables can facilitate a more comprehensive and in-depth understanding of the intrinsic relationship between the two variables. For example, Fang et al. (2025) assert that consistent physical exercise can accelerate the accumulation of psychological capital, which may, in turn, positively influence career decision outcomes. Multiple studies have shown that engaging in physical exercise can significantly enhance the psychological capital of college students (Lin et al., 2025; Wei et al., 2024; Zhao et al., 2025). According to Morgan (2018), physical exercise has enormous potential to positively influence an individual’s psychological capital. Further examining the core components of psychological capital, Li et al. (2022) found that college students who participated in physical activities displayed significantly higher levels of self-efficacy than those who did not participate. Physical exercise has a significant positive predictive effect on self-efficacy (Han et al., 2022; Zhang et al., 2024). To et al. (2022) discovered that individuals who adhered to physical exercise guidelines exhibited higher levels of psychological resilience compared to individuals who did not engage in adequate physical exercise. Kim et al. (2016) found that physical exercise significantly predicts optimism, while Zhang et al. (2023) noted a similar positive predictive effect of physical exercise on hope. Psychological capital is a measurable, developable, and manageable positive psychological state that facilitates individuals to showcase efficient performance on tasks through their personal and professional growth (Luthans et al., 2007). This construct consists of four main components: self-efficacy, optimism, resiliency, and hope (Luthans et al., 2007). This fundamental definition is supported by a systematic review conducted by Li et al. (2023), which highlights the role of psychological capital as a key personal resource for college students. The review links psychological capital to critical elements within the career decision-making context, including enhanced academic engagement, reduced stress, and the practice of adaptive coping strategies. By examining these dimensions of psychological capital alongside existing research on career decision-making, it becomes evident that there could be a negative correlation between psychological capital and CDMD. For example, Chen et al. (2025) found that psychological capital is negatively related to the CDMD among college students. Studies by Pang et al. (2021) and Shin and Kelly (2015) both show that psychological resilience is negatively associated with CDMD. Studies by Firdaus and Arjanggi (2020) and Pignault et al. (2023) both show that self-efficacy is negatively related to CDMD. Magnano et al. (2015) found that optimism is positively related to effective decision-making styles. In (2016) found that hope and career decision self-efficacy are positively associated. Additionally, strengthening psychological capital also helps shorten the transition period from school to work for college students, which facilitates the development of positive coping strategies, enhances job satisfaction, and alleviates employment anxiety (Baluku et al., 2021; Belle et al., 2021). Therefore, it is believed that both psychological capital as a whole and the four-dimensional structure of psychological capital may be negatively correlated with CDMD.

Considering these findings, psychological capital can be considered as a mediating variable between physical exercise and CDMD. Specifically, this can also be examined through the lens of Conservation of Resources Theory (Hobfoll, 1989). As a “resource builder,” physical exercise fosters psychological capital and subsequently buffers against CDMD by offsetting decision-related uncertainty and stress. Establishing a model that positions psychological capital as the mediator will provide a vital theoretical framework for understanding the influence of physical exercise on CDMD.

After establishing that psychological capital may serve as a mediating mechanism linking physical exercise to CDMD, it is necessary to delve deeper into the potential indirect relationship between psychological capital and CDMD. Employment attitudes, defined as psychological tendencies and behavioral choices during the employment process, encompass cognitive and behavioral characteristics related to career choice, job search, and work commitment (Li, 2022). Research consistently demonstrates robust connections among employment attitudes, psychological capital, and CDMD. For instance, psychological capital has been identified as a positive predictor of employment attitudes (Choi and Park, 2019), enabling individuals to maintain proactive job-seeking behaviors and thereby enhancing employment prospects (Georgiou and Nikolaou, 2018; Fernández-Valera, 2023). Similarly, Mohanty (2010, 2012) observed that positive attitudes and optimism increase the likelihood of securing employment. Collectively, these findings support the proposition that psychological capital is positively associated with employment attitudes. Sustaining a positive outlook further improves access to diverse job opportunities. Such proactive attitudes manifest in behaviors like actively seeking job information, tracking recruitment trends, and formulating clear career plans (Li, 2022), which help individuals navigate competitive job markets and mitigate decision-making difficulties arising from information asymmetry (Liu, 2009). While a positive employment attitude may not directly guarantee job placement, it empowers individuals to overcome obstacles in the employment process, thereby increasing their chances of securing employment.

**Figure 1:**
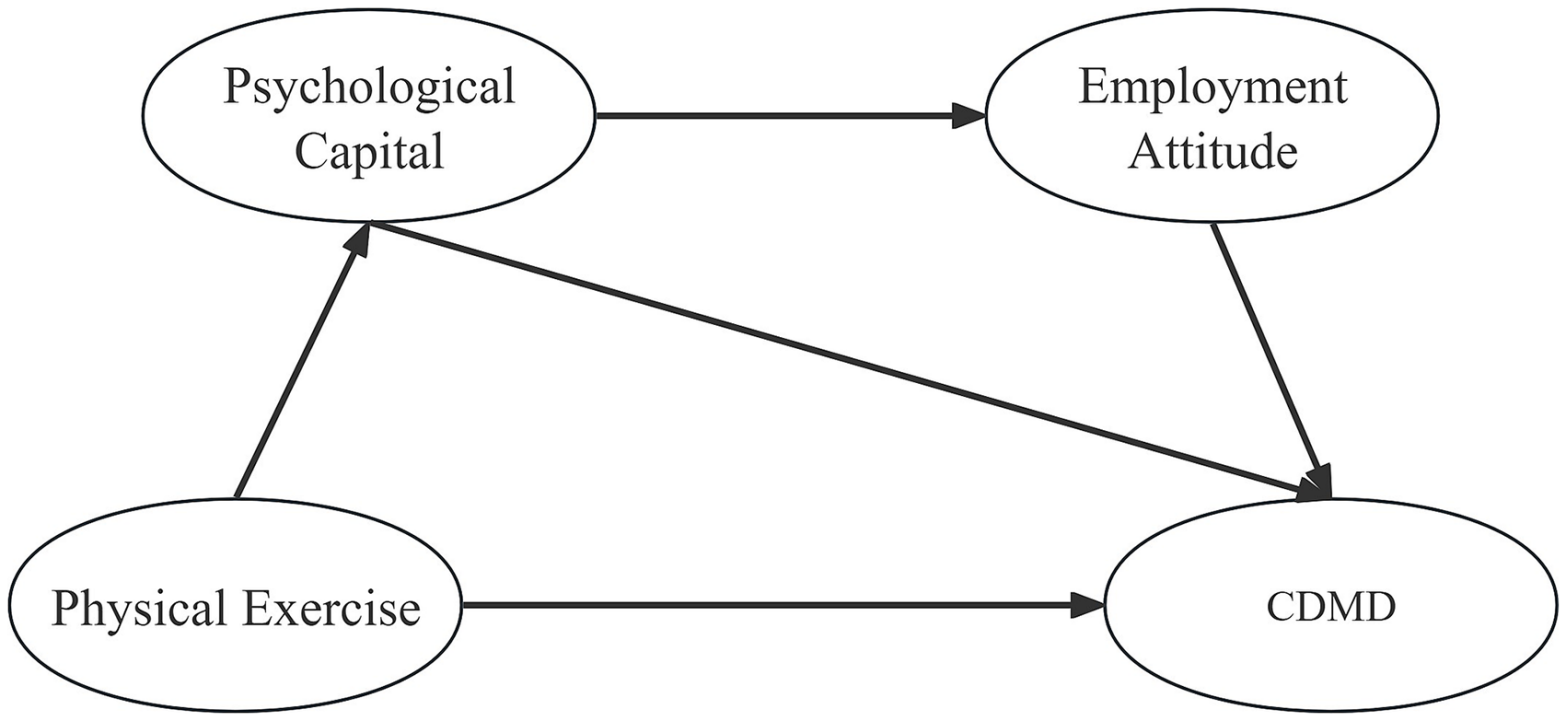
The proposed conceptual model.

Based on the above review of the relationships among physical exercise, psychological capital, career attitude, and CDMD, a gap remains in the research on the association between physical exercise and CDMD. This study aims to provide new theoretical insights and practical directions for alleviating CDMD among college students by exploring this relationship. In summary, this study constructs a hypothetical research model (Figure 1) that illustrates the hypothetical relationships among the variables. Furthermore, recognizing the differences between urban and rural college students regarding access to job information, living environments, and lifestyles—and existing studies that have identified significant differences in career decision-making between these two groups—this study incorporates urban–rural difference as a moderating variable (Guo, 2016). The four specific research hypotheses formulated are as follows:

*Hypothesis 1 (H1)*: There is a significant positive association between physical exercise and the CDMD among college students.

*Hypothesis 2 (H2)*: Psychological capital serves as a mediator between physical exercise and CDMD.

*Hypothesis 3 (H3)*: Both psychological capital and employment attitudes have a chain mediating effect on the relationship between physical exercise and CDMD.

*Hypothesis 4 (H4)*: The pathways taken by the research model in this study are moderated by urban–rural differences.

## 2 Methods

### 2.1 Measures

To assess the key variables such as physical exercise, psychological capital, CDMD, and employment attitude, validated scales adapted to the Chinese college student context were used. All scales employed a 5-point Likert response format, and their reliability (measured by Cronbach’s *α*) and construct validity (assessed through confirmatory factor analysis, CFA) were evaluated in the current sample.

#### 2.1.1 Physical exercise scale

The physical exercise measurement scale developed by Zou (2017) was used to measure the physical exercise of college students. The scale comprises five items, one of which is: “No matter how busy I am, I can always find time to exercise.” Responses were measured on a five-point Likert scale, and the scale was unidimensional. A higher total score from these five items indicates a greater level of physical exercise and stronger persistence in engaging in physical exercise. This scale, developed for Chinese college students, has demonstrated good reliability and validity in previous studies. In this study, the Cronbach’s alpha coefficient for this scale was 0.908. The reference indicators for confirmatory factor analysis were as follows: *χ*^2^/df = 4.679, TLI = 0.988, GFI = 0.993, RMSEA = 0.061, CFI = 0.995, SRMR = 0.012.

#### 2.1.2 Psychological capital scale

The psychological capital scale used in this study was originally developed by Luthans et al. (2007) and was modified by Shi (2014). It comprises 18 items, such as: “I believe I can speak or present information to a group of classmates” and “I usually handle stress in my studies calmly.” The scale uses a five-point Likert scale and is divided into four dimensions: self-efficacy, optimism, hope, and resiliency. A higher level of psychological capital is indicated by a higher overall score. The scale’s Cronbach’s alpha coefficient in this investigation was 0.948. The alpha coefficients for each dimension were as follows: optimism = 0.868; self-efficacy = 0.885; resilience = 0.848; and hope = 0.884. For the confirmatory factor analysis, the reference indicators were *χ*^2^/df = 3.284, GFI = 0.953, TLI = 0.969, RMSEA = 0.048, CFI = 0.974, SRMR = 0.026.

#### 2.1.3 Career decision-making difficulties (CDMD) scale

The Chinese version of the CDMD Scale was developed by Du and Long (2006), building on Gati et al.’s (1996) original, adapted to the Chinese cultural context. Later, Liu (2008) revised the scale. This scale employs a five-point Likert scale comprising 16 items, such as: “I have read books or articles about career planning” and “I have participated in training or social practice that aids career planning.” The scale comprises four dimensions: career self-exploration, career information exploration, career planning exploration, and career goal exploration. Given that a higher score on the original scale indicates a lower level of CDMD, and considering that subsequent analyses would require repetitive logical conversions-which could lead to confusion for readers-all item scores in this study were reverse-coded. This adjustment ensures that a higher total score now corresponds to a higher level of CDMD, thereby aligning the scores with the core meaning of “CDMD.” In this study, the scale demonstrated a Cronbach’s alpha coefficient of 0.937. The alpha coefficients for each dimension were as follows: career goal exploration at 0.862; exploration of career information at 0.865; career planning exploration at 0.769; and career self-exploration at 0.801. The indicators for confirmatory factor analysis were as follows: *χ*^2^/df = 5.086, TLI = 0.944, GFI = 0.938, RMSEA = 0.064, CFI = 0.955, SRMR = 0.034.

#### 2.1.4 Employment attitude scale

The Employment Attitude Scale was developed by Li (2022). This scale employs a five-point Likert scale comprising 12 items, such as: “I consistently track school recruitment updates” and “I proactively seek employment information.” The scale comprises four dimensions: positivity, openness, rationality, and focus. A higher total score indicates a more positive attitude toward employment. During the reliability testing phase of this study, the rationality dimension exhibited an overall reliability score of 0.604. A further examination of the values revealed that the Corrected Item-Total Correlation (CITC) coefficient for Item 3 was 0.228, which is significantly lower than the reference criterion of 0.4. Furthermore, the Cronbach’s alpha coefficient after removing this item was 0.738, exceeding the original alpha of 0.604 (Wu, 2010). After thorough discussion among the research team and following the recommendations from Wu (2010), it was decided to remove this item. As a result, the final employment attitude scale achieved an overall Cronbach’s alpha coefficient of 0.884. The alpha coefficients for each dimension were as follows: positivity at 0.779; openness at 0.619; rationality at 0.738; and focus at 0.855. The indicators for confirmatory factor analysis were as follows: *χ*^2^/df = 7.862, CFI = 0.947, GFI = 0.945, RMSEA = 0.083, TLI = 0.923, SRMR = 0.043.

### 2.2 Sample

This study employed non-probability convenience sampling techniques to conduct an online questionnaire survey among college students at two universities in Guizhou Province, China. The study has been approved by the Ethics Committee for Sports Science at Shandong Sport University. The legal rights and interests of the survey respondents were completely respected and safeguarded, and the entire questionnaire survey procedure complied with the principles of voluntariness and anonymity. A total of 1,134 questionnaires were collected, with 132 deemed invalid due to identical or overly consistent responses. This resulted in 1,002 valid questionnaires for data analysis, yielding an effective response rate of 88%. The demographic information is summarized in Table 1.

**Table 1:** Distribution of demographic variables.

Characteristics	Categories	Number of people (%)
Gender	Male	541(54%)
	Female	461(46%)
Major	Science and engineering	499(50%)
	Humanities and social sciences	503(50%)
Class standing	Freshmen	377(38%)
	Sophomore	347(35%)
	Junior	220(22%)
	Senior	58(6%)
Students’ place of origin	Urban	513(51%)
	Rural	489(49%)

### 2.3 Procedure

Data were collected through an online questionnaire, with all items designated as mandatory to prevent missing responses. Participants first reviewed an informed consent form that outlined the study’s purpose and ethical guarantees, and they completed the questionnaire only if they agreed to participate. The entire process adhered to the principles of informed consent, anonymity, and voluntariness to protect participants’ legal and ethical rights.

### 2.4 Data analysis

The descriptive statistics were conducted using normality tests based on skewness and kurtosis indices, reliability tests based on Cronbach’s *α* coefficient, correlation analysis, and regression analysis, using SPSS 26.0. Since this study used an online questionnaire, all of the items were made mandatory in order to guarantee that no data were omitted. Given that demographic variables might influence college students’ levels of physical exercise, employment attitudes, psychological capital, and CDMD, these variables were included as covariates in the regression analysis model to control for potential confounding effects and ensure accurate results. The research hypotheses were validated using AMOS 24.0 software by constructing a structural equation model (SEM). There is no universal standard within academia regarding the number of resamples for the Bootstrap resampling method. However, Efron (1988) recommended that when constructing confidence intervals, the number of resamples should be at least 1,000. Building on Zhu’s (1997) observation that the greater the number of resamples, the more accurate the estimates, we utilized the Bootstrap method using 5,000 random resamples to construct the required confidence intervals. The mediating impact was considered significant if it did not include 0 in its 95% confidence interval. Several indicators and predetermined criteria were used to assess the model’s goodness-of-fit, including: *χ*^2^/df (<3), CFI (<0.9), GFI (<0.9), RMSEA (<0.08), TLI (<0.9), and SRMR (<0.05). Additionally, this study used multi-group analysis to investigate the moderating influence of urban–rural disparities because urban and rural locations represent categorical variables. Furthermore, inter-group difference tests were performed for every path using the Bootstrap method; if a significant difference was found in a particular path, it meant that urban–rural differences had a moderating influence on that path.

## 3 Results

### 3.1 Common method bias

In this study, self-reported data were used, which might have led to common method bias (Podsakoff et al., 2003). A single-factor confirmatory factor analysis method was employed to test common method bias. First, all items and variables were combined to construct a second-order latent variable model. The model fit indices were: *χ*^2^/df = 2.983, GFI = 0.861, TLI = 0.922, SRMR = 0.041, RMSEA = 0.045, CFI = 0.927. Then, all items were combined to construct a single-factor model, with the following fit indices: *χ*^2^/df = 9.133, GFI = 0.588, TLI = 0.681, SRMR = 0.075, RMSEA = 0.090, CFI = 0.694. A comparison revealed that the overall fit indices for the single-factor model were inadequate. As a result, it was concluded that the data used in this study did not show significant common method bias (Bae, 2021).

### 3.2 Correlation tests among variables

The results of the normality test reveal that the absolute skewness values of all variables are less than 2, and the absolute kurtosis values are less than 7. This indicates that the data in this study are normally distributed (Kim, 2013). As illustrated in Table 2, a significant positive correlation was observed among the three variables: physical exercise, psychological capital, and employment attitude, with correlation coefficients of *r* = 0.553 (*p* < 0.01), *r* = 0.366 (*p* < 0.01), and *r* = 0.654 (*p* < 0.01). Conversely, CDMD displayed a significant negative correlation with physical exercise, psychological capital, and employment attitude, yielding correlation coefficients of *r* = −0.484 (*p* < 0.01), *r* = −0.757 (*p* < 0.01), and *r* = −0.705 (*p* < 0.01). These significant correlations among the variables justify further data analysis.

**Table 2:** Results of correlation analysis between variables.

Variables	Physical exercise	Psychological capital	CDMD	Employment attitude
Physical exercise	1			
Psychological capital	0.553^**^	1		
CDMD	−0.484^**^	−0.757^**^	1	
Employment attitude	0.366^**^	0.654^**^	−0.705^**^	1
Mean	2.891	3.370	2.659	3.602
Standard deviation	0.913	0.630	0.645	0.593

**p < 0.01.

### 3.3 Regression analysis

To ensure the accuracy of the regression analysis results, gender, class standing, major, and students’ place of origin were included as covariates in the model. The results of the regression analysis are illustrated in Table 3: Physical exercise has a significant positive correlation with psychological capital (*β* = 0.583, *p* < 0.001); physical exercise and CDMD (*β* = −0.103, *p* < 0.001) have a significant negative correlation; psychological capital and employment attitude have a significant positive correlation (*β* = 0.643, *p* < 0.001); psychological capital has a significant negative correlation with CDMD (*β* = −0.465, *p* < 0.001); employment attitude has a significant negative correlation with CDMD (*β* = −0.356, *p* < 0.001); and physical exercise has no significant correlation with employment attitude (*β* = 0.016, *p* > 0.05).

**Table 3:** Multiple linear regression analysis.

Variables	Psychological capital		Employment attitude		CDMD	
	Beta (*β*)	*t*	Beta (*β*)	*t*	Beta (*β*)	*t*
Gender	0.117	4.206***	0.046	1.799	−0.040	−2.018*
Major	−0.05	−1.825	0.004	0.143	0.055	2.863**
Class standing	−0.033	−1.207	0.046	1.848	−0.068	−3.565***
Students’ place of origin	−0.064	−2.414*	−0.034	−1.427	0.049	2.656**
Physical exercise	0.583	21.346***	0.016	0.529	−0.103	−4.425***
Psychological capital			0.643	22.187***	−0.465	−17.039***
Employment attitude					−0.356	−14.581***
CDMD						
*R* ^2^	0.323		0.434		0.665	
∆*R*^2^	0.320		0.430		0.663	
*F*	95.172***		127.009***		282.075***	

Beta (β), standardized coefficients; *R*^2^, R square; ∆*R*^2^, Adjusted R Square; *p < 0.05, **p < 0.01, ***p < 0.001.

### 3.4 Mediation analysis

**Figure 2:**
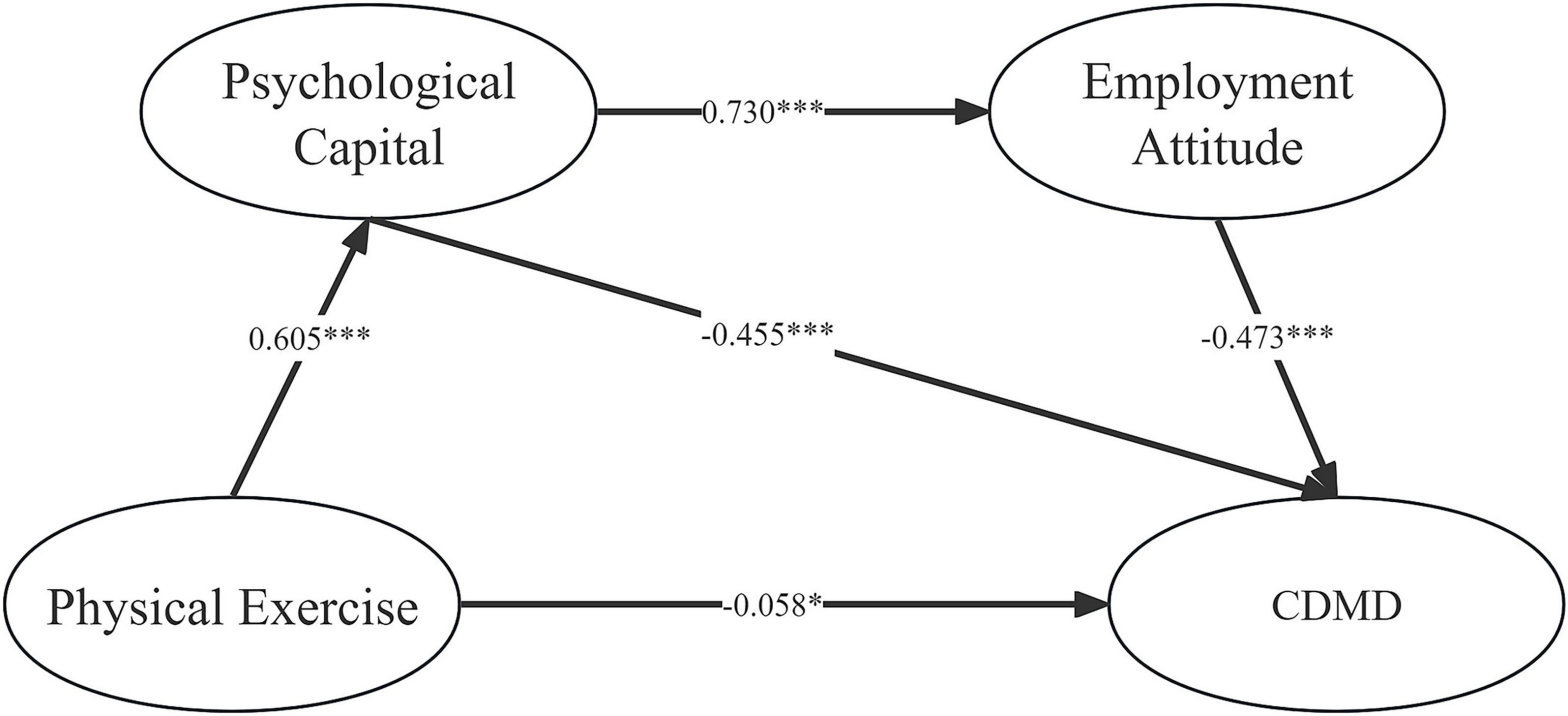
Chain mediation model (normalized). ***p < 0.001, *p < 0.05.

The model constructed based on the research hypothesis is shown in Figure 2. The model fits well, with specific fitting indicators as follows: *χ*^2^/df = 6.277, GFI = 0.915, RMSEA = 0.073, TLI = 0.940, CFI = 0.950, SRMR = 0.04. As depicted in Table 4, there is a significant negative association between physical exercise and CDMD, with a direct effect of −0.058 (95% CI: −0.117, −0.003), accounting for 11% of the total effect. This finding provides support for Hypothesis 1. Furthermore, as illustrated in Table 4, psychological capital functions as a mediator in the relationship between physical exercise and CDMD, manifesting an indirect effect of −0.276 (95% CI: −0.348, −0.209), which accounts for 51% of the total effect. Consequently, Hypothesis 2 was also supported. The findings of this study demonstrate a chained mediating effect between physical exercise and CDMD, with an indirect effect of −0.209 (95% CI: −0.260, −0.166), explaining 38% of the total effect. This finding lends support to Hypothesis 3. CDMD falls by 0.543 standard deviations for every standard deviation increase in physical exercise, according to the overall impact value of −0.543. Further supporting the idea that psychological capital is the primary mediating variable is the fact that 89% of path effects are mediated by psychological capital overall.

**Table 4:** Proportion of mediating effects (normalized).

Path	Effect size	BootSE	Bootstrap 95% CI		Proportion %
			Lower	Upper	
Physical activity → Psychological capital → CDMD	−0.276	0.036	−0.348	−0.209	51%
Physical activity → Psychological capital → Employment attitude → CDMD	−0.209	0.024	−0.260	−0.166	38%
Direct effect	−0.058	0.029	−0.117	−0.003	11%
Total effect	−0.543	0.029	−0.600	−0.485	100%

### 3.5 Moderating effect test

The fit indices of the two groups, urban and rural, were as follows: Urban group: *χ*^2^/df = 3.953, CFI = 0.947, RMSEA = 0.076, TLI = 0.937, SRMR = 0.039; Rural group: *χ*^2^/df = 4.309, CFI = 0.934, RMSEA = 0.082, TLI = 0.922, SRMR = 0.051. Both models demonstrated good fit, providing a solid foundation for subsequent analyses. As shown in Table 5, regarding the significance of each path in the urban and rural group models, only the direct effect in the rural group was non-significant, with a direct effect value of −0.015 (95% CI: −0.1, 0.076). All other paths were significant. When comparing path differences between the two groups, only the path “physical exercise → Psychological capital → CDMD” showed a significant difference, with an estimated value of −0.176 (95% CI: −0.3, −0.063). Thus, it was concluded that urban–rural differences exert a moderating effect on the “physical exercise → Psychological capital → CDMD” path.

**Table 5:** Test of urban–rural differences.

Path (group)		Bootstrap 95% CI		
		Estimate	Lower	Upper
Rural	R1: Direct effect	−0.015	−0.1	0.076
	R2: Physical activity → Psychological capital → CDMD	−0.377	−0.501	−0.264
	R3: Physical activity → Psychological capital → Employment attitude → CDMD	−0.188	−0.277	−0.122
Urban	U1: Direct effect	−0.081	−0.16	−0.003
	U2: Physical activity → Psychological capital → CDMD	−0.201	−0.286	−0.121
	U3: Physical activity → Psychological capital → Employment attitude → CDMD	−0.211	−0.28	−0.16
Comparison of differences	diff2(R1-U1)	0.066	−0.019	0.157
	diff1(R2-U2)	−0.176	−0.3	−0.063
	diff3(R3-U3)	0.024	−0.065	0.09

## 4 Discussion

### 4.1 The relationship between physical exercise and CDMD

Results revealed a significant negative association between physical exercise and CDMD, which is consistent with the findings of Fang et al. (2025) and Kim (2019). However, on employing the Bootstrap method, which has higher statistical power and lower requirements for sample distribution assumptions (Efron and Tibshirani, 1994), it was found that although the direct association between physical exercise and CDMD reached the level of significance, it was close to the critical value. One tentative explanation for this marginal significance could relate to the operationalization of the CDMD scale in this study, which was assessed based on individuals’ “existing career goals or plans” and “ongoing behaviors or thoughts” related to career decisions (Du and Long, 2006; Liu, 2008). In contrast, Fang et al. (2025) noted that career decision self-efficacy focused on an individual’s confidence in completing future career decision tasks, suggesting a potential discrepancy in construct measurement that may have influenced effect sizes. Another provisional factor contributing to the marginal significance might be the tendency of Chinese college students to overestimate their personal abilities (Song, 2025). This tendency could lead them to equate academic performance with overall career competence, often overlooking gaps in social and teamwork skills. Such cognitive bias may have attenuated the direct effect of physical exercise on alleviating CDMD. Additionally, when comparing rural and urban models, the direct effect was significant in the urban model but not in the rural model, despite no statistically significant difference between the two effects. This observed pattern can be provisionally attributed to two potential factors: first, rural college students may have engaged in more lifelong physical activity compared to their urban peers, potentially creating a ceiling effect for exercise’s additional impact; second, limited availability of physical activity resources in rural areas could have weakened exercise’s positive influence. Importantly, this tentative interpretation lacks robust theoretical and empirical support, so it must be confirmed by future research, and citations of this finding should be made with caution. Furthermore, the observation that the significance level of the direct association approached the critical value solely when psychological capital was used as the core mediating variable demonstrates that the accumulation of psychological capital might serve as an essential pathway through which physical exercise interventions influence career decisions. It suggests that physical exercise must first be transformed into components of psychological capital, such as resilience and self-efficacy, in order to effectively reduce decision-making challenges. This finding addresses a notable gap in the literature regarding the theoretical underpinnings of the connection between physical exercise and difficulties in career decision-making.

While the correlation between the two variables approaches the critical value, it also indicates the potential influence of additional mediating variables. This suggests that the observed significance may be attributed to the mediating effects of other relevant factors. For example, individuals dissatisfied with their body image often attribute their failure to uncontrollable physical factors or a perceived lack of autonomy, stemming from ineffective dietary and exercise habits. These factors are likely to facilitate a pessimistic attitude toward career choices (Kim and Kwon, 2024). One of the key benefits of physical exercise is its ability to improve body image, which in turn can boost individuals’ confidence in making future career decisions (Woodrow-Keys, 2006; Kim and Kwon, 2024). However, a notable limitation of these studies is that their participants were exclusively female, which means that their applicability to male populations has yet to be verified. Furthermore, participation in group sports can enhance social skills, which may also influence career choices. Individuals often evaluate the social skills necessary for specific careers against their own abilities to assess their fit for those roles (Fang et al., 2025). Moreover, engaging in exercise has been shown to reduce employment-related anxiety, helping individuals maintain a positive mindset when facing challenges in the career decision-making process (Kim et al., 2020). Additionally, it should be clarified that the variables discussed above are conceptualized as having the potential to function as mediating variables and are provided as a reference for future research.

### 4.2 The mediating effects of psychological capital

As mentioned earlier, the association between physical exercise and CDMD is mediated by psychological capital. Specifically, there is a positive correlation between physical exercise and psychological capital, aligning with previous research findings (Deng and Wang, 2024; Luo et al., 2025; Xu et al., 2025). Within the context of physical exercise, accomplishing goals, overcoming obstacles, sustaining a positive mindset, and enhancing self-discipline all contribute to the development of an individual’s psychological capital (Fang et al., 2025; Lee et al., 2022; Morgan, 2018). Psychological capital is negatively associated with CDMD, which aligns with previous research findings (Firdaus and Arjanggi, 2020; In, 2016; Magnano et al., 2015; Zhou et al., 2024). By integrating the psychological capital-mediated pathway with four key issues that Chinese college students face in career decision-making-namely, biases in career information acquisition, self-perception biases, biases in career goal-setting, and biases in external environment perception-it is argued that this psychological capital-mediated pathway is crucial for addressing Chinese college students’ career cognition issues among these students (Song, 2025). From the perspective of biases in career information acquisition and external environment perception, a lack of psychological capital leads college students to be unmotivated in exploring career options and understanding employment trends. This often manifests as low self-efficacy, inadequate psychological resilience, lack of confidence when facing challenges, fear of failure, a persistent pessimistic attitude toward employment issues, weak commitment to career goals, and a lack of adaptability to adjust those goals (Luthans et al., 2007).

When it comes to self-perception biases and career goal-setting, college students frequently set idealized career goals. Physical exercise is likely to help students in objective evaluation of the alignment between their personal abilities and occupational requirements, thus bridging the gap between ideal career aspirations and reality—thereby reducing CDMD. Therefore, it can be inferred that physical exercise may reduce CDMD by enhancing individuals’ psychological capital. Another notable finding of this study is that urban–rural differences moderated this pathway, with the rural group contributing more to alleviating CDMD. On one hand, this may be due to the relatively scarce mental health resources available to rural college students, making physical exercise a crucial pathway for enhancing their psychological capital. On the other hand, urban college students may have greater access to mental health services and diverse social support, reducing their reliance on a single channel (i.e., physical exercise) for psychological well-being. Furthermore, the regression analysis results indicate a lack of a significant relationship between physical exercise and employment attitudes. This could be attributed to the fact that the association between physical exercise and employment attitudes may only be realized through mediating mechanisms, such as self-efficacy and psychological resilience. Future studies may incorporate additional mediating variables to unravel the mechanism underlying their relationship. In the overall model, the combined proportion of path effects mediated by psychological capital accounts for 89%. Consequently, the development of the overall model hinges on the crucial mediating role of psychological capital, highlighting its importance as a core mediating variable.

According to the findings of this study, university physical education courses or physical exercise programs could attempt to alleviate college students’ difficulties in making career decisions by implementing modular activities that improve self-efficacy, hope, resilience, and optimism. More specifically, self-efficacy can be improved through incremental skill training, such as setting stepwise sports goals or taking on team leadership roles. Additionally, individuals’ psychological resilience can be bolstered by creating high-pressure competitive scenarios. Alleviating anxiety and stress through exercise further enhances students’ self-regulation abilities and helps them maintain an optimistic mindset. Furthermore, career development courses for college students can also be optimized through the integration of structured physical activities. This model addresses challenges such as inadequate learning motivation, minimal classroom participation, and a dull environment often found in single-theory instruction. Enhancing experiential and interactive learning improves learning outcomes and facilitates the achievement of teaching objectives.

This integration of physical exercise and career education effectively addresses the limitations of current career guidance in Chinese universities. Existing studies reveal that university career guidance centers and courses are often underutilized due to outdated content and vague information (Tan et al., 2025). By incorporating physical activities into career development courses, universities can enhance student engagement while addressing cognitive biases such as low self-efficacy and idealized goals at their source. For example, group sports activities in career courses can improve teamwork skills and build psychological capital, which, in turn, supports students in understanding the social skills required for various careers and reduces CDMD caused by skill mismatches.

Overall, this study developed a theoretical model that integrates physical exercise, psychological capital, and CDMD. This model serves as a foundation for future empirical research on using physical exercise as an intervention for college students experiencing CDMD. Its key value lies in its capacity to lower CDMD levels by enhancing psychological capital and utilizing the learning transfer effect from sports contexts, while simultaneously promoting physical and mental health development. Furthermore, this model can be integrated into physical education and vocational education courses in higher education. This research not only broadens the application of psychological capital theory in career development but also provides theoretical guidance and practical pathways for reforming vocational education and physical education course systems in colleges and universities.

### 4.3 Chain mediating effects

The findings indicate that psychological capital and employment attitudes play a chain mediating role in the relationship between physical exercise and CDMD. Notably, psychological capital is positively correlated with employment attitudes, which is consistent with the findings of Choi and Park (2019). From the perspective of the Theory of Planned Behavior (Ajzen, 1991), enhancing psychological capital activates the core components of this theory. Specifically, improving self-efficacy, a key dimension of psychological capital, directly strengthens perceived behavioral control and encourages proactive job-seeking behaviors, such as submitting internship applications and pursuing skill certifications. Additionally, hope and optimism foster a positive attitude toward employment by redefining challenges as opportunities; for instance, viewing competitive industries as catalysts for growth instead of obstacles. These efforts ultimately enhance individuals’ competitiveness in meeting entry-level job requirements and industry standards (Wang and Yuan, 2021). This proactive behavior, driven by attitude, plays a vital role in encouraging college students to pursue and achieve their desired career goals (Li, 2022). The chain mediating pathway outlined in this study addresses two key issues faced by Chinese college students in their career decision-making. First, regarding biases in information acquisition, a strong psychological capital fosters a positive employment attitude, which motivates students to proactively seek formal information channels, such as university career guidance centers and corporate internships. This proactive approach helps reduce CDMD caused by information asymmetry. Second, regarding cognitive lag in industry and career awareness, proactive career preparation behaviors driven by employment attitudes, such as participating in internships and learning skills relevant to their desired industry, enable students to update their understanding of industry trends. This, in turn, helps narrow the gap between their own abilities and job requirements, further alleviating CDMD.

From a long-term development perspective, it not only enhances initial job stability but also establishes an essential psychological and behavioral foundation that supports future career adaptability and long-term success. Furthermore, while this study has effectively outlined the chain-mediated path of “psychological capital → employment attitude,” it is important to recognize that focusing exclusively on this pathway falls short. The factors influencing career decision-making challenges are complex and multifaceted. For instance, some Chinese college students have vague career goals: they tend to have only general aspirations (e.g., “to find a good job”) rather than specific plans for industry positioning or skill development. Consequently, their goals are easily swayed by external factors. Future research could investigate the inclusion of the “career goal clarity” variable in the mediation model. Additionally, given the regional differences in employment markets across China, future studies should consider regional factors to test the cross-regional applicability of the model, ensuring that the findings more accurately reflect the diverse career decision-making contexts of Chinese college students.

### 4.4 Limitations

Although this study provides empirical support for understanding the mechanism linking physical exercise to CDMD, several limitations remain: (1) the cross-sectional design limits our ability to establish the causal direction between the variables. Future research should aim to verify the temporal effects and causal chains through longitudinal tracking or experimental interventions. (2) While the correlation between physical exercise and CDMD exhibits statistical significance, its effect size approaches the critical threshold for significance. Additionally, existing theoretical frameworks and empirical studies have not yet adequately supported this association. Future research is advised to integrate multi-dimensional theoretical frameworks and empirical evidence to conduct in-depth verification of the underlying mechanism of this relationship. (3) The model did not account for other potential predictor variables, such as career identity and social support, which may affect the completeness of the mechanism. Future research is encouraged to include additional associated variables and utilize multi-group analysis or cross-lagged models to explore the dynamic interactions among multiple variables. (4) This study employed a non-probability sampling method to administer questionnaire surveys among Chinese college students. Additionally, the sample was characterized by a low proportion of senior-class students (in terms of class year) and a high proportion of rural students. These factors may limit the generalizability of the conclusions. Future studies are recommended to adopt alternative sampling methods and validate these findings across diverse cultural contexts and populations, thereby enhancing the generalizability of the results. (5) While the structure of psychological capital may go beyond the current four-factor framework, the current results and practical recommendations are based only on mainstream theories and measuring methodologies (Luthans et al., 2007). Therefore, the fundamental mediating function of psychological capital needs to be revalidated as theories develop and measures improve.

## 5 Conclusion

This study reveals that physical exercise is associated with college students’ CDMD through both the single mediating effect of psychological capital and the chain mediating effect of psychological capital and employment attitudes. Physical exercise demonstrates significant potential for alleviating CDMD among college students. By constructing a chain mediation model, this study addresses the research gap regarding the mechanisms linking physical exercise to CDMD, laying a theoretical foundation for future empirical studies on using physical exercise as an intervention for CDMD. This model represents an innovative attempt to explore multiple pathways for alleviating college students’ CDMD, while also expanding the application of the psychological capital theory in the field of career development. The model established in this study can be integrated into physical education and vocational education curricula in higher education, thereby enhancing college students’ career decision-making capabilities. It provides a theoretical basis and practical pathways for innovating relevant courses. Future studies can build on this research to further optimize intervention strategies that utilize physical exercise to alleviate CDMD, addressing existing limitations in the research.

## Data Availability

The raw data supporting the conclusions of this article will be made available by the authors, without undue reservation.

## References

[ref1] Ajzen I. (1991). The theory of planned behavior. Organ. Behav. Hum. Decis. Process. 50, 179–211. doi: 10.1016/0749-5978(91)90020-T

[ref2] Amir T. Gati I. (2006). Facets of career decision-making difficulties. Br. J. Guidance. Couns. 34, 483–503. doi: 10.1080/03069880600942608

[ref3] Anghel E. Gati I. (2019). The associations between career decision-making difficulties and negative emotional states. J. Career Dev. 48, 537–551. doi: 10.1177/0894845319884119

[ref4] Bae B. R. (2021). Structural equation modeling with Amos 27. Seoul: Chungram Publishing.

[ref5] Baluku M. M. Mugabi E. N. Nansamba J. Matagi L. Onderi P. Otto K. (2021). Psychological capital and career outcomes among final year university students: the mediating role of career engagement and perceived employability. Int. J. Appl. Posit. Psychol. 6, 55–80. doi: 10.1007/s41042-020-00040-w

[ref6] Belle M. A. Antwi C. O. Ntim S. Y. Affum-Osei E. Ren J. (2021). Am i gonna get a job? Graduating students’ psychological capital, coping styles, and employment anxiety. J. Career Dev. 49, 1122–1136. doi: 10.1177/08948453211020124

[ref7] Chen W. Guo S. Y. Guo Z. H. (2025). The impact of parental career deficit on college students' career decision-making difficulties: the chain mediating effect of psychological capital and career exploration. J. Xian Jiaotong Univ. Med. Sci. 46, 574–579.

[ref8] Choi Y. J. Park G. J. (2019). The impact of the youth's positive psychological capital to reemployment attitudes. J. Korea Academia-Ind. Coop. Soc. 20, 40–46. doi: 10.5762/KAIS.2019.20.10.40

[ref9] Deng Y. R. Wang X. L. (2024). The impact of physical activity on social anxiety among college students: the chain mediating effect of social support and psychological capital. Front. Psychol. 15:1406452. doi: 10.3389/fpsyg.2024.1406452, PMID: 38957885 PMC11217649

[ref10] Du R. Long L. R. (2006). A study on career decision-making difficulties questionnaire for undergraduate students. Chin. J. Clin. Psychol. 3, 237–239.

[ref11] Efron B. (1988). Bootstrap confidence intervals: good or bad? Psychol. Bull. 104, 293–296. doi: 10.1037/0033-2909.104.2.293

[ref12] Efron B. Tibshirani R. J. (1994). An introduction to the bootstrap. 1st Edn. New York: Chapman & Hall/CRC.

[ref13] Fang Y. Xu T. T. Ye M. S. Li C. Q. (2025). The relationship between physical activity and career decision-making self-efficacy in Chinese college students: the mediating roles of self-control and social anxiety. Front. Psychol. 16:1541211. doi: 10.3389/fpsyg.2025.1541211, PMID: 39944038 PMC11815557

[ref14] Fernández-Valera M.-M. (2023). Psychological capital and job search: a systematic literature review and agenda for future research. Int. J. Res. Vocat. Educ. Train. 10, 68–89. doi: 10.13152/IJRVET.10.1.4

[ref15] Firdaus W. Arjanggi A. (2020). Self-efficacy and career decision making difficulties in senior high school students. Indig. J. Ilm. Psikol. 5, 141–150. doi: 10.23917/indigenous.v5i2.8941

[ref16] Fouad N. Cotter E. W. Kantamneni N. (2009). The effectiveness of a career decision-making course. J. Career Assess. 17, 338–347. doi: 10.1177/1069072708330678

[ref17] Gati I. Krausz M. Osipow S. H. (1996). A taxonomy of difficulties in career decision making. J. Couns. Psychol. 43, 510–526. doi: 10.1037/0022-0167.43.4.510

[ref18] Gati I. Kulcsár V. (2021). Making better career decisions: from challenges to opportunities. J. Vocat. Behav. 126:103545. doi: 10.1016/j.jvb.2021.103545

[ref19] Georgiou K. Nikolaou I. (2018). The influence and development of psychological capital in the job search context. Int. J. Educ. Vocat. Guid. 19, 391–409. doi: 10.1007/s10775-018-9385-2

[ref20] Gu X. Tang M. Chen S. Montgomery M. L. (2020). Effects of a career course on Chinese high school students’ career decision-making readiness. Career Dev. Q. 68, 222–237. doi: 10.1002/cdq.12233

[ref21] Guo L. (2016). Research on the influence of social support on the college students’ career decision-making self-efficacy. Mod. Educ. Manag. 3, 112–116.

[ref22] Han S. S. Li B. Wang G. X. Ke Y. Z. Meng S. Q. Li Y. X. . (2022). Physical fitness, exercise behaviors, and sense of self-efficacy among college students: a descriptive correlational study. Front. Psychol. 13:932014. doi: 10.3389/fpsyg.2022.932014, PMID: 35910985 PMC9335150

[ref23] Hobfoll S. E. (1989). Conservation of resources: a new attempt at conceptualizing stress. Am. Psychol. 44, 513–524. doi: 10.1037/0003-066X.44.3.513, PMID: 2648906

[ref24] In H. (2016). Acculturation and hope as predictors of career decision self-efficacy among Korean international undergraduate students. J. Career Dev. 43, 526–540. doi: 10.1177/0894845316633784

[ref25] Jia Y. Hou Z. Zhang R. (2025). Career decision-making difficulties and mental health: the joint moderations of proactive coping and future time perspective. J. Career Assess. 1, 1–17. doi: 10.1177/10690727251343022

[ref26] Kim H. Y. (2013). Statistical notes for clinical researchers: assessing normal distribution (2) using skewness and kurtosis. Restor. Dent. Endod. 38, 52–54. doi: 10.5395/rde.2013.38.1.52, PMID: 23495371 PMC3591587

[ref27] Kim H. S. (2018). Effects of the physical self-efficacy and body image on career decision level of university students. J. Sport Leisure. Stud. 74, 309–318. doi: 10.51979/KSSLS.2018.11.74.309

[ref28] Kim H. S. (2019). The effects of university students’ participation in sports activities on the decision level of progress. J. Sport Leisure. Stud. 78, 301–308. doi: 10.51979/KSSLS.2019.10.78.301

[ref29] Kim S. Y. Jeon S. W. Lee M. Y. Shin D. W. Lim W. J. Shin Y. C. . (2020). The association between physical activity and anxiety symptoms for general adult populations: an analysis of the dose-response relationship. Psychiatry Investig. 17, 29–36. doi: 10.30773/pi.2019.0078, PMID: 31856560 PMC6992859

[ref30] Kim E. Kwon M. (2024). Body dissatisfaction, perceived gender discrimination, belief in a just world, and career-choice pessimism in Korean female college students: a moderated mediation model. Int. J. Adv. Counselling 46, 620–636. doi: 10.1007/s10447-024-09562-3

[ref31] Kim J. Lee S. Chun S. Han A. Heo J. (2016). The effects of leisure-time physical activity for optimism, life satisfaction, psychological well-being, and positive affect among older adults with loneliness. Ann. Leis. Res. 20, 406–415. doi: 10.1080/11745398.2016.1238308

[ref32] Komarraju M. Swanson J. Nadler D. (2014). Increased career self-efficacy predicts college students’ motivation, and course and major satisfaction. J. Career Assess. 22, 420–432. doi: 10.1177/1069072713498484

[ref33] Lam M. Santos A. (2018). The impact of a college career intervention program on career decision self-efficacy, career indecision, and decision-making difficulties. J. Career Assess. 26, 425–444. doi: 10.1177/1069072717714539

[ref34] Lee K. Bae H. Jang S. (2022). Effect of exercise combined with natural stimulation on korean college students’ concentration and positive psychological capital: a pilot study. Healthcare 10:673. doi: 10.3390/healthcare10040673, PMID: 35455850 PMC9030325

[ref35] Li J. (2022). Research on cultivation and optimization of college students’ employment value in the new era (dissertation). Changchun, China: Northeast Normal University.

[ref36] Li R. Che Hassan N. Saharuddin N. (2023). Psychological capital related to academic outcomes among university students: a systematic literature review. Psychol. Res. Behav. Manag. 16, 3739–3763. doi: 10.2147/PRBM.S421549, PMID: 37705849 PMC10497058

[ref37] Li X. N. Liu M. Yu H. F. Zhang Z. J. He Z. H. (2022). The influence of sports on proactive personality and academic achievement of college students: the role of self-efficacy. Front. Psychol. 13:943347. doi: 10.3389/fpsyg.2022.943347, PMID: 36118457 PMC9476941

[ref38] Lin Y. He M. J. Zhou W. Q. Zhang M. H. Wang Q. Chen Y. Y. . (2025). The relationship between physical exercise and psychological capital in college students: the mediating role of perceived social support and self-control. BMC Public Health 25:581. doi: 10.1186/s12889-025-21856-8, PMID: 39939931 PMC11823263

[ref39] Liu Y. (2008). The research on career decision-making difficulty of the undergraduate students in independent college (dissertation). Wuhan, China: Central China Normal University.

[ref40] Liu Z. (2009). Attitude of persuasion theory and adjusting employment expectation and deviation of college students. Res. Educ. Dev. 19, 67–70.

[ref41] Luo X. Y. Liu H. Q. Sun Z. Y. Wei Q. Zhang J. H. Zhang T. C. . (2025). Gender mediates the mediating effect of psychological capital between physical activity and depressive symptoms among adolescents. Sci. Rep. 15:10868. doi: 10.1038/s41598-025-95186-5, PMID: 40158032 PMC11954977

[ref42] Luthans F. Youssef C. M. Avolio B. J. (2007). Psychological capital: Developing the human competitive edge. Oxford: Oxford University Press.

[ref43] Magnano P. Paolillo A. Giacominelli B. (2015). Dispositional optimism as a correlate of decision-making styles in adolescence. SAGE Open 5, 215824401559200–215824401559212. doi: 10.1177/2158244015592002

[ref44] Mohanty M. S. (2010). Effects of positive attitude and optimism on employment: evidence from the US data. J. Socio-Econ. 39, 258–270. doi: 10.1016/j.socec.2009.12.004

[ref45] Mohanty M. S. (2012). Effects of positive attitude and optimism on wage and employment: a double selection approach. J. Socio-Econ. 41, 304–316. doi: 10.1016/j.socec.2012.01.004

[ref46] Morgan H. (2018). Enhancing social mobility within marginalized youth: the accumulation of positive psychological capital through engagement with community sports clubs. Sport Soc. 21, 1669–1685. doi: 10.1080/17430437.2017.1409725

[ref47] Otu M. Onyishi C. (2024). A culturally nuanced exploration of adolescent career decision-making and mental health challenges. J. Adv. Guid. Couns. 5, 87–104. doi: 10.21580/jagc.2024.5.2.24959

[ref48] Pang L. M. Wang X. Liu F. Fang T. T. Chen H. R. Wen Y. (2021). The relationship between college students’ resilience and career decision-making difficulties: the mediating role of career adaptability. Psychology 12, 872–886. doi: 10.4236/psych.2021.126053 PMC9145158 35624459

[ref49] Pignault A. Rastoder M. Houssemand C. (2023). The relationship between self-esteem, self-efficacy, and career decision-making difficulties: psychological flourishing as a mediator. Eur. J. Investig. Health Psychol. Educ. 13, 1553–1568. doi: 10.3390/ejihpe13090113, PMID: 37754452 PMC10529372

[ref50] Podsakoff P. M. MacKenzie S. B. Lee J. Y. Podsakoff N. P. (2003). Common method biases in behavioral research: a critical review of the literature and recommended remedies. J. Appl. Psychol. 88, 879–903. doi: 10.1037/0021-9010.8.8.5.879 14516251

[ref51] Shi B. M. (2014). The effect of proactive personality on college students' creativity: The mediation role of psychological capital and creative motivation (dissertation). Hangzhou, China: Zhejiang University.

[ref52] Shin Y. J. Kelly K. R. (2015). Resilience and decision-making strategies as predictors of career decision difficulties. Career Dev. Q. 63, 291–305. doi: 10.1002/cdq.12029

[ref53] Song Y. (2025). Challenges faced in promoting college students' correct professional cognition. Labor Soc. Secur. 16, 145–146.

[ref54] Tan G. Wu F. Mai Y. (2025). Research on the teaching reform of “industry-academia-research-innovation” collaborative education oriented to enhance students' employment competence. Ind. Innov. 17, 196–198.

[ref55] To Q. G. Vandelanotte C. Cope K. Khalesi S. Williams S. L. Alley S. J. . (2022). The association of resilience with depression, anxiety, stress and physical activity during the COVID-19 pandemic. BMC Public Health 22:491. doi: 10.1186/s12889-022-12911-9, PMID: 35279118 PMC8917786

[ref56] Wang Z. Fan C. Niu J. (2023). Predicting effects of career adaptability and educational identity on the career decision-making of Chinese higher vocational students. Int. J. Educ. Vocat. Guidance 25, 111–130. doi: 10.1007/s10775-023-09591-1, PMID: 37360275 PMC10060938

[ref57] Wang G. Yuan D. Y. (2021). An empirical research on the influencing factors of higher vocational students' employment attitudes. Contemp. Vocat. Educ. 3, 30–37.

[ref58] Wang L. Zhai Y. Y. Sun Q. C. (2024). Enhancing career adaptability in college students: a tai chi-based sports intervention study. Front. Psychol. 15:1455877. doi: 10.3389/fpsyg.2024.1455877, PMID: 39399269 PMC11467863

[ref59] Wei X. L. Lai Z. J. Tan Z. W. Ou Z. Y. Feng X. Y. Xu G. Q. . (2024). The effect of physical activity on depression in university students: the mediating role of self-esteem and positive psychological capital. Front. Psychol. 15:1485641. doi: 10.3389/fpsyg.2024.1485641, PMID: 39380753 PMC11458536

[ref60] Willner T. Gati I. Guan Y. (2015). Career decision-making profiles and career decision-making difficulties: a cross-cultural comparison among US, Israeli, and Chinese samples. J. Vocat. Behav. 88, 143–153. doi: 10.1016/j.jvb.2015.03.007

[ref61] Woodrow-Keys E. (2006). The relationship between body image and career decision-making self-efficacy in college female athletes and non-athletes (Dissertation). Huntington, WV, USA: Marshall University.

[ref9001] World Health Organization. (2010). Global recommendations on physical activity for health. Geneva, Switzerland: World Health Organization Press.

[ref62] Wu M. L. (2010). Questionnaire statistical analysis practice-spss operation and application. Chongqing: Chongqing University Press.

[ref63] Xu A. A. Luo X. B. Zhou G. Z. Lu C. F. (2025). Physical exercise and aggressive behavior in rural left-behind children: the mediating roles of psychological capital and self-control. BMC Psychol. 13:438. doi: 10.1186/s40359-025-02736-7, PMID: 40275397 PMC12023496

[ref64] Zhang G. Feng W. X. Zhao L. Y. Zhao X. H. Li T. J. (2024). The association between physical activity, self-efficacy, stress self-management and mental health among adolescents. Sci. Rep. 14:5488. doi: 10.1038/s41598-024-56149-4, PMID: 38448518 PMC10917799

[ref65] Zhang X. P. Wang D. X. Li F. (2023). Physical exercise, social capital, hope, and subjective well-being in China: a parallel mediation analysis. Int. J. Environ. Res. Public Health 20:303. doi: 10.3390/ijerph20010303 PMC9819114 36612625

[ref66] Zhao H. B. You Q. Shi L. (2025). The relationship between physical activity and college students’ sense of security: the chain-mediated role of self-esteem and psychological capital. Sci. Rep. 15:19392. doi: 10.1038/s41598-025-02484-z, PMID: 40461507 PMC12134343

[ref67] Zhou A. Liu J. Xu C. Jobe M. C. (2024). Effect of social support on career decision-making difficulties: the chain mediating roles of psychological capital and career decision-making self-efficacy. Behav. Sci. 14:318. doi: 10.3390/bs14040318, PMID: 38667114 PMC11047401

[ref68] Zhu W. (1997). Making bootstrap statistical inferences: a tutorial. Res. Q. Exerc. Sport 68, 44–55. doi: 10.1080/02701367.1997.10608865, PMID: 9094762

[ref69] Zou R. (2017). Study on the relationship between sport involvement and subjective well-being of college students-based on a sample survey of college students in shanghai (dissertation). Shanghai, China: Shanghai University of Sport.

